# A Health Impact Assessment Framework for Assessing Vulnerability and Adaptation Planning for Climate Change

**DOI:** 10.3390/ijerph111212896

**Published:** 2014-12-12

**Authors:** Helen Brown, Jeffery Spickett, Dianne Katscherian

**Affiliations:** School of Public Health, World Health Organization Collaborating Centre for Environmental Health Impact Assessment, GPO Box U1987, Perth, WA 6845, Australia; E-Mails: j.spickett@curtin.edu.au (J.S.); diannekat@iprimus.com.au (D.K.)

**Keywords:** climate change, adaptation, health impact assessment, vulnerability

## Abstract

This paper presents a detailed description of an approach designed to investigate the application of the Health Impact Assessment (HIA) framework to assess the potential health impacts of climate change. A HIA framework has been combined with key climate change terminology and concepts. The fundamental premise of this framework is an understanding of the interactions between people, the environment and climate. The diversity and complexity of these interactions can hinder much needed action on the critical health issue of climate change. The objectives of the framework are to improve the methodology for understanding and assessing the risks associated with potential health impacts of climate change, and to provide decision-makers with information that can facilitate the development of effective adaptation plans. While the process presented here provides guidance with respect to this task it is not intended to be prescriptive. As such, aspects of the process can be amended to suit the scope and available resources of each project. A series of working tables has been developed to assist in the collation of evidence throughout the process. The framework has been tested in a number of locations including Western Australia, Solomon Islands, Vanuatu and Nauru.

## 1. Introduction

Human health and well-being are inextricably linked to climate via a large number of environmental, social and economic variables [1,2]. It is clear that change to temperature, rainfall, extreme events and sea-level will have impacts on health and that current strategies to address these may prove ineffective. Without appropriate adaptation, risks to the health and well-being of billions of people will increase [3]. 

The scope and extent of climate-related health impacts will be strongly influenced by location and it is paramount that strategies to adapt to climate change are formulated at the national, regional and local level. The complexity and size of this task, as well as high levels of uncertainty and incomplete evidence, present an incredible challenge [4,5]. Nevertheless, it is clear that such an undertaking is necessary to ensure that we are properly prepared for the impacts imposed by climate change now and in the future. 

The World Health Organisation defines Health Impact Assessment (HIA) as “a combination of procedures, methods and tools by which a policy, programme or project may be judged as to its potential effects on the health of the population, and the distribution of those effects within the population” [6]. Despite differences in the form of HIAs, a general consensus has emerged regarding the procedural steps [7,8]. HIA has been acknowledged as a valuable tool for identifying and assessing the multiple pathways that link climate change to human health [9,10,11].

In addition to the procedural steps typically used in HIA, the framework presented here includes practical tools that have been developed to provide guidance on the specific issue of climate change. The framework also incorporates climate change terminology and concepts. This guidance should encourage stakeholders to tackle what may otherwise seem a daunting task. In addition, the framework and working tables enable a comparison of findings between locations and periodic updating of assessments. 

## 2. The Process

**Figure 1:**
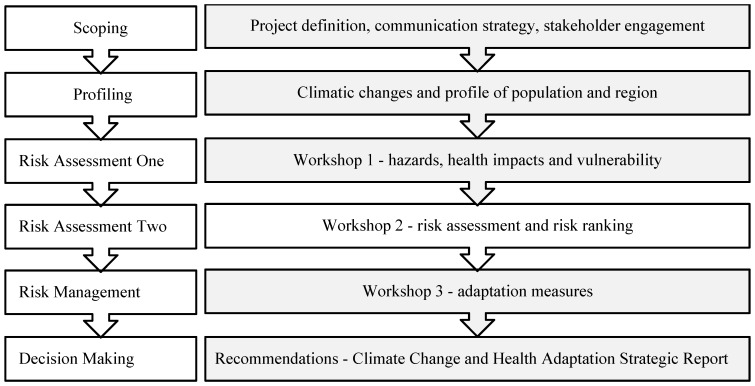
HIA framework for climate change vulnerability and adaptation assessment.

An overview of the process, based on a Health Impact Assessment (HIA) framework, is provided in Figure 1. The process requires the establishment of a Project Team and the active involvement of expert stakeholders from health and other sectors during three workshops. A series of working tables have been created to assist with various tasks within each step of the process. This paper provides a brief outline of each step and examples of the working tables completed as part of a HIA of climate change in Western Australia (WA) [10,12]. Application of the framework in several locations has led to the development of guide entitled “Climate Change, Vulnerability and Health: A Guide to Assessing and Addressing the Health Impacts” which provides additional examples [13].

### 2.1. Step 1—Scoping

The scoping step establishes and identifies the key concepts of the project including; clear administrative procedures; a preliminary consideration of links between climate change and determinants of health and; factors affecting vulnerability to climate-related health effects.

This step includes:

- the establishment of the Project Team and Terms of Reference
- development of a communication strategy
- development of a stakeholder engagement strategy

The Terms of Reference for the Project Team should establish the key factors for consideration such as:

- a shared understanding of the definition of health and of climate-related health impacts
- spatial and temporal boundaries of the HIA
- roles and responsibilities of members
- decision making processes within the Team including agreement on methodologies for the project
- resource requirements (e.g., funding, time, budgets, staff)
- time lines for activities
- final output (e.g., recommendations for decision makers)

Working table 1 (Table 1) lists variables potentially affected by climate change that can affect human health and well-being. The table categorises the potential health impacts into eight main groups and is based on the WHO definition of health as “a dynamic state of complete physical, mental and social wellbeing and not merely the absence of disease or infirmity” [14]. This step helps to establish a common understanding amongst the Project Team of the range of potential health impacts that may be considered during the process. It is likely that resources may not enable a full consideration of all listed impacts or that some impacts may not be relevant for specific locations. The Project Team will already have an indication of the climate-related impacts of most concern for the population being considered and this may result in a short-list of impacts to be considered in the HIA. 

**Table 1:** Working table 1: Potential health-related impacts to consider.

**Direct Effects of Extreme Climate Events** ▪ Physical Injury/Death from extreme climate events	
**Indirect Effects of Climate Change**	
**Environmental factors** ▪ Air quality ▪ Water quality ▪ Soil quality ▪ Food contamination ▪ Pathogens ▪ Vector-borne disease factors /Vermin ▪ Broader environmental issues (CO_2_ emissions) ▪ Food Production—crops and animals ▪ Visual amenities (green space, coastline)	**Ecological factors** ▪ Loss of habitat ▪ Impacts on plant diseases, pests, weeds ▪ Physical changes to land—coastline, rivers, erosion, landslides ▪ Changes to groundwater levels ▪ Flora and fauna—change in distribution
**Socio-economic factors** ▪ Employment ▪ Occupational health and safety ▪ Social networks ▪ Local business ▪ Economic issues ▪ Crime ▪ Housing ▪ Population changes	**Psychosocial factors** ▪ Mental health—control over life, stress, anxiety ▪ Community well-being ▪ Social conflict
**Lifestyle factors** ▪ Exercise ▪ Diet ▪ Health behaviour ▪ Alcohol/drugs	**Technological factors** ▪ Accidents (mechanical, chemical, *etc*.) ▪ Fire, explosions ▪ Waste treatment
**Services** ▪ Resource availability ▪ Access to emergency services ▪ Routine access to health services (primary/secondary) ▪ Routine access to other services (schools, shops, transport)	**Insert other factors as required** ▪ ▪

#### 2.1.1. Vulnerability

Vulnerability is defined as “the degree to which a system is susceptible to or unable to cope with, adverse effects of climate change” [15]. In terms of HIA, vulnerability is strongly linked to the principle of equity [8,16]. An understanding of vulnerability helps to ensure that adaptation strategies target vulnerable groups and reduce potential inequities with respect to the health burden of climate change [17]. 

Exposure, sensitivity and adaptive capacity are the three fundamental elements that contribute to overall vulnerability and it is critical that the Project Team and stakeholders have a shared understanding of these elements [14]. The pathway between a climatic variable and the subsequent health impact often involves multiple steps and vulnerability can manifest at any point in that pathway [18]. 

Understanding the health impact pathway is a powerful tool for identifying points of vulnerability as well as opportunities for adaptation. For example, vulnerability to health effects of heatwaves can stem from; differences in exposure patterns based on occupational and social variables; the sensitivity of individuals to extreme heat; the capacity of the energy sector to meet peak demand during heatwaves and; differences in community and individual capacity to implement adaptation strategies. 

There are multiple factors that affect the three main elements of vulnerability [12]. It is recommended that vulnerability is considered in terms of regional, economic, social and infrastructure aspects. An early understanding of the elements influencing vulnerability highlights the importance of collaboration between multiple sectors and helps to inform the communication and stakeholder engagement strategies. 

#### 2.1.2. Communications and Stakeholder Engagement Strategies

Development of a communications strategy should enable members of the Project Team to communicate activities, reduce the potential for disagreement and enhance participation of stakeholders in workshops and other activities. The strategy should also consider the different forms of communication for both internal and external stakeholders and establish key responsibilities, reporting requirements and deadlines over the course of the project.

Stakeholder engagement is critical as many of the potential health impacts are linked to effects that occur outside the jurisdiction of the health sector. The stakeholder engagement strategy is based on seven key steps as developed by the Department of Health in Western Australia [19]. The list of potential health impacts in working table 1 and an understanding of vulnerability help to guide the stakeholder engagement strategy. The fundamental issues for consideration are incorporated in working table 2 but for the purposes of this paper are listed below:

- Who should take responsibility for consultation?
- Who are the key stakeholder groups at the national, regional and local level?
- How are the needs of and consultation with vulnerable groups to be addressed?
- Can representation for the needs of particular groups be obtained and is this useful?
- What should the outcomes of the consultation be used for?
- Are there different timeframes required for consultation, communication and dissemination of information?

### 2.2. Step 2—Profiling of Climate, Region and Population

The profiling step is undertaken by the Project Team and involves; the collection of background information on climate change; key characteristics of the natural and built environment and; demographic and baseline data on the health status of the population and existing health services. The extent of this data will be influenced by the decisions made during the scoping phase.

Information on potential climate changes for the location and chosen timeframe is sourced from government departments, meteorological services and other publically available sources such as IPCC reports. Previous Project Teams have selected the year 2030 or 2050 however this can be amended to suit the specific project [12,20,21,22]. The basic climate data should include projections for:

- Gradual changes—temperature, rainfall and sea level
- Extreme climatic events—Heat waves, cold snaps, tropical cyclones, storm surges, floods, droughts and bushfires

Basic characteristics of the natural or built environment that make areas more vulnerable to certain health impacts should also be included (e.g., vulnerability of low-lying coastal areas to sea-level increase or urban areas to extreme heat). An understanding of basic population demographics such as age distribution and life expectancy should be included to identify vulnerable groups. The current health status and leading causes of mortality and morbidity, particularly in relation to existing climate-sensitive diseases such as malaria and asthma, should be compiled. Key information from the scoping and profiling steps should be provided to the stakeholders. 

### 2.3. Step 3—Risk Assessment One

Risk assessment aims to: (i) identify variables with links to health that may be affected by climate and; (ii) establish the extent and type of health impacts that may occur as a result of the climate-related changes in the selected scenario. These steps are conducted in two separate workshops. As discussed previously, the range of climate-related hazards to consider can be determined during the scoping phase. 

#### Step 3—Workshop 1

Workshop 1 is typically held over a day and includes stakeholders from a broad range of sectors including health, emergency services, environment, indigenous affairs, planning, housing, development, water, energy, transport, and agriculture. The main output of the day is the completion of working tables 3–5. 

#### Hazard Identification

The identification of potential climate-related health hazards is guided by three main categories:

- Biophysical—air, water, food. Includes environmental-related illness related to air, water and food quality and vector-borne diseases
- Social—economy, lifestyle, housing, workforce, population displacement, psychosocial factors and community services
- Infrastructure –energy, transport, built environment, telecommunications, water, waste

Participants are assigned to groups based on their area of expertise. Group size of 6–8 is recommended. If numbers permit, more than one group can be formed for each category or the different elements of each category can be divided. Each group identifies the health-related hazards in their category associated with the climate projections outlined in the Profiling step. Although the categories help to focus discussion it is likely that overlap between categories will occur. Examples of climate-related hazards for the Biophysical category have been combined in Table 2, but during the workshop a separate sheet is provided for each of the climatic variables (*Sheets 1 to 4*) [12].

**Table 2:** Working table 3: Potential climate-related hazards.

Climatic Variable	Biophysical Category
	*Insert List of Relevant Health-Related Hazards*
**Temperature** *(Sheet 1)*	Ground-level ozone likely to increase with higher summer temperatures
**Rainfall** *(Sheet 2)*	Reductions in rainfall—reduced water quality, water stress.
**Sea-level** *(Sheet 3)*	Mosquito breeding sites may be affected
**Extreme Events** *(Sheet 4)*	Heatwaves—direct heat-related effects, air quality Bushfire—air quality, potential contamination of water supplies, impact on food production

With four sheets per category, at least 12 working tables are completed. This step typically takes a few hours and many climate-related hazards are identified. At the end of the session each group reports their key findings in a plenary session. This step ensures that participants are aware of information from other groups that may have relevance for subsequent discussions across other categories. 

#### Health Impacts and Vulnerability Assessment

The second part of the workshop utilises the information recorded in working table 3. The potential environmental hazards identified by the Biophysical, Social and Infrastructure groups are transferred to working table 4. The direct and indirect health impacts that may occur as a result of these hazards are identified and the factors influencing vulnerability to each health effect are discussed. The key elements of exposure, sensitivity and adaptive capacity, as well as the suggested categories (regional, economic, social and infrastructure) are used to guide the discussion. An understanding of the health impact pathway is critical in this step and must be underpinned by an appropriate level of knowledge and expertise of the workshop participants. Examples of responses in the Biophysical category related to temperature increase and heatwaves are provided in Table 3. 

**Table 3:** Working table 4: Health impacts of biophysical category.

Climate Variable	Health Hazards & Impacts		Vulnerability (Exposure, Sensitivity, Adaptive Capacity)				Evidence/ Uncertainty
Gradual Changes	Hazards *(Tansfer from working table 3)*	Health Impacts *Direct & indirect*	Regional	Economic	Social	Infrastructure	
***Temperature increase***	Ground-level ozone likely to increase	Respiratory and cardio-vascular effects, including increase in mortality, hospitalisations and doctor visits.	Exposure likely to be higher in urban areas	-	Exposure tends to be higher outdoors → lifestyle and occupational factors may increase exposure.	Flow-on effects to health sector. May be heightened during heatwaves.	Link between ozone levels and temperature, and health effects of ozone exposure are well-established.
	Increase in aeroallergens	Asthma			Sensitive groups—existing respiratory conditions, including asthma.		Effect on aeroallergens is complex and uncertain.
**Extreme Events** ***Heatwaves***	Exposure to extreme heat	Heat-related illnesses	Areas with higher temperatures. Urban areas due to urban heat island effect. Higher proportion of sensitive groups in some regions (elderly, isolated).	Low income groups—lower adaptive capacity and affected more by energy costs incurred during heatwaves. Food producers who may face crop losses, possible impact on tourism.	Elderly, isolated, pre-existing medical conditions. Low adaptive capacity—low income groups, homeless Higher exposure—certain occupations or lifestyles	Power cuts caused by high levels of peak demand. Damage to transport systems. Flow on effects to industry. Increased demand on health services.	Link between exposure to heat and health is well-established. Possible synergistic effects of exposure to heat and air pollutants should be considered.

Excerpts from [12].

#### Current Management Practices and Limitations

Current management practices for each of the health impacts in working table 4 are recorded in working table 5 (Table 4). It may also be useful to record brief notes in this table during the previous step as current management actions are often mentioned at that point. The group considers the effectiveness of these responses for the selected year taking into consideration climate change projections and other major demographic changes. 

**Table 4:** Working table 5: Current management practices and limitations.

Impact Type	Current Management Practices	Potential Limitations in 2030	Sector Column
Air quality—range of respiratory effects	Air Quality Management Program Medical treatment	Air Quality Management Plan requires updating Lack of resources	Environment Health Transport
Heatwaves - Direct heat-related effects	State Emergency Management Committee All West Australian Reducing Emergencies (AWARE)	More extreme events—will be more demand Ageing population—larger vulnerable group Lack of specific heat-wave response plan Lack of preparedness/education especially in remote indigenous communities Impact of energy blackouts on vulnerable groups	Emergency Services, Health, Energy, Indigenous Affairs

Excerpts from [12].

The volume of information obtained from Workshop 1 is substantial. Post-workshop, the Project Team is collates the information and confirms key sources of evidence. This is likely to involve ongoing collaboration with stakeholders and in some cases further data collection from local health services or the literature. A summary of Workshop 1 outcomes should be disseminated to all participants, who are given an opportunity to provide further comments prior to the second workshop. 

### 2.4. Step 4—Risk Assessment Two: Workshop 2

The objective of Workshop 2 is to ascertain the level of risk associated with the health impacts identified in the first workshop. This is typically a half-day workshop with fewer participants who are selected for their expertise in health risk assessment. Prior to the workshop, the Project Team sorts the impacts from working table 4 into eight categories listed below [12,20]. These impacts are recorded in working table 6.

- Extreme Events
- Temperature Increase and Related Changes
- Water-borne Disease and Water Quality
- Vector-borne diseases
- Air Quality
- Food-borne diseases
- Food Production
- Social Impact/Community Lifestyle—e.g., Dislocation, Mental Health

Health sector participants (who are likely to have attended the first workshop) are assigned to a group that considers one or more of the above categories. Participants should be provided with the Workshop One summary, a copy of the working table 6 that their group will complete and a description of the risk assessment process. An outline is provided below, but readers are referred to the full guidelines for a complete description [13].

The health consequences of each impact and the likelihood of them occurring are assessed using a predetermined scale. The level of risk is determined by entering the consequence and likelihood rankings into a risk assessment matrix. The assessment requires expert judgement based on the available evidence and assumes that decisions regarding the level of risk are based on:

- the climate change projections and year as outlined by the Project Team
- a consideration of current management practices for each health impact
- the level of excess or additional risk linked to climate change

Health consequences are assessed using a five level qualitative scale based on the severity and extent of the impact. If sufficient evidence is available it is possible that a quantitative assessment can be undertaken. In this case, readers are referred to other sources for quantitative health consequence scales [22,23]. The likelihood of consequences occurring also uses a 5 level scale based on previously published likelihood scales developed for climate change assessments [24]. 

Each group discusses the evidence and comes to an agreement with respect to the consequence and likelihood levels, recording the rationale used. If consensus is not possible this is noted. The risk level is determined from a simple 5x5 risk assessment matrix with risk levels ranging from low to extreme. Table 5 provides several examples from the Extreme Events group in the Western Australian study [12]. 

**Table 5:** Working table 6: Risk assessment table for extreme events.

Impact	Consequence	Likelihood	Risk	Rationale/Further Evidence.
Heat-related health effects during heatwaves	Catastrophic	Very Likely	Extreme	Strong evidence of link between heatwaves and health. Studies indicate increase in multiple heat-related fatalities due to climate change in Perth in 2030 [25]. Ageing population will increase size of vulnerable population.
Bushfires	Very High	Likely	High	Drier and hotter conditions in WA are likely to increase risk of fires. Possible fatalities and injuries, exposure to high particulate levels, significant psychosocial and socioeconomic costs. Vulnerable populations—bushfire prone areas, South-West WA.

The risk rankings are compiled, health impacts sorted from the highest to the lowest level of risk and collectively discussed in a plenary session. Given the qualitative nature of the assessment, it is possible that a comparison of risk levels from different groups may identify apparent anomalies in the final risk ranking. In these cases, the evidence is compared, and any final adjustments to risk ranking are made. 

### 2.5. Step 5—Risk Management or Adaptation

The risk management step of HIA is also referred to as adaptation, as this term is routinely used in relation to management of climate change impacts. The Project Team collates all of the information from Workshop 2 and the final list of risk levels. As shown in Table 6, descriptions of management actions for each risk level, including the level of community acceptability, helps to determine which impacts will be carried through to final workshop [21]. For example it may be determined that subsequent steps will only consider health impacts assessed as a high or extreme risk. 

**Table 6:** Management of climate-sensitive health risks.

Risk Levels	Description of Management Action
Extreme	Risks require urgent attention at the most senior level and cannot simply be accepted by the community
High	Risk are the most severe that can be accepted by the community and need planned action
Medium	Risks can be expected to be part of normal circumstances but maintained under review by appropriate sectors
Low	Risks will be maintained under review but it is expected that existing controls will be sufficient and not further action will be required to treat them unless they become more severe

#### Workshop 3—Adaptation Measures

This step is conducted in a final half-day or full day workshop. The Project Team must ensure that participants include those with the knowledge to identify appropriate adaptation measures and to influence decision-making with respect to such measures. Participants are provided with an overview of first two workshops and a summary of the final workshop format prior to attending. 

Participants are assigned to groups focusing on one or more of the eight categories from Workshop 2 as well as an additional category of “General Principles and Adaptation Measures”. Groups consider a list of potential adaptation strategies compiled prior to the workshop and also identify additional strategies relevant to their setting. The current status of each strategy in the study area is rated as: A—adequate; I—inadequate; D—developing and/or; N—Not in place. An example is provided in Table 7. This rating includes a consideration of vulnerability for each impact. Participants provide suggestions on how current management strategies can be upgraded or implemented and identify the sectors that are likely to be involved. 

**Table 7:** Working table 7: Potential adaptation strategies for extreme events (excluding heatwaves).

Categories of Adaptation	What Is Our Capacity *—In General and for Vulnerable Regions and Groups?		Suggestions for Implementation or Upgrading	Sectors Involved
**1. Legislative or Regulatory**				
• Cost sharing mechanisms for compensation and adaptation initiatives.	N	Only private insurance	Appropriate upgrades of procedures and assessments as climate change projections and assessments dictate.	Treasury, Insurance Planning, Housing Consumer Affairs, Emergency Services
• Regulations for minimum building standards to withstand extreme events in vulnerable regions.	A	Amend regulations as required		
• Regulations regarding fire management, property management to reduce risk of injuries.	A	Amend regulations as required		
• Mid to long-term strategies for land use planning that accounts for likely impacts	N			
**2. Public Education & Communication**			Wider community engagement needed Modern communication should be available to all (e.g., broadband)	Communication Health Local Government
• Improvement in communicating risks of extreme events to vulnerable regions and groups.	I/D	Continued improvement and greater investment required.		
• Education of measures to reduce risk of damage or injuries	D	Coordination with Federal government is required.		
• Evaluation of the effectiveness of educational materials.	I			
**3. Surveillance and Monitoring**			Access to GP data Up to date environmental and population forecasts Monitoring needs upgraded as required	Health, Planning, Environment, Climate Research Emergency Services Insurance industry
• Standardization of information collected after disasters to more accurately measure morbidity and mortality.	I	Long-term follow up is not adequate Hospital morbidity data is okay		
• Evaluation of responses and health outcomes of extreme events.	I			
**4. Ecosystem Intervention**			Upgrade as needed Mostly mitigation but needs to address adaptation and prediction	Environment Agriculture Research Water, Planning
• Monitor the effects of altered land use on vulnerability to extreme weather events.	A	-		
**5. Infrastructure Development**			Emergency system needs to expand to cope with more frequent and more severe extreme events	Emergency Services, Health, Local Gov’t Planning, Water Energy, Transport
• Create or enhance emergency management—communication, preparation, training, volunteer recruitment, emergency response coordination, resource allocation.	I/D	North-west seen as vulnerable		
• Mapping of potential risks from extreme events—location of hazardous facilities, vulnerable properties/people.	I/D	All understood to some extent		
• Land use planning and management to minimize impacts from cyclones, flooding and fire (protective structures, controlled burning).	I/D	Need to highlight the necessity to Treasury to upgrade infrastructure as necessary.		
**6. Technological or Engineering**				
• Improvement of systems to provide early and accessible warning to the populations most likely to be affected.	D/A	Systems are in place The main issues are access to information and the community response to early warning systems.	Expand resources as required	Climate Research Building Health
• Modification of building codes for structures in vulnerable areas.				
**7. Health Intervention**				
• Improved training programmes and information on emergency management.	A	Enhance responses to rural and regional areas	Continue development	Health Emergency Services
**Research/ Information**				
• Regional assessments of vulnerability to extreme events.	All either I or D	-	-	Whole of Government Health Research Climate Local Gov’ tIndigenous
• Regional identification of vulnerable communities and individual.				
• Evaluate effectiveness of early warning systems.				
• Further development of early warning systems—tropical cyclones, fires, droughts.				
• Modelling of affected regions				

Notes: * A = adequate, I = inadequate, D = developing, N = not in place. Excerpt from [12]

Selected examples from the WA study of potential adaptation categories related to the direct physical impacts of extreme tropical cyclones, storms, floods and bushfires are provided in Table 7 [12]. As heatwaves posed a higher level of risk than other extreme events they were considered separately in the WA study. Similar adjustments to the suggested categories can be made for the specific locations as deemed necessary. Readers are referred to the WA study [12] and the guide [13] for examples of potential adaptation strategies in the other categories. Each group presents their results in a plenary session. This is a critical step as it disseminates information across traditional sectors and highlights potential synergies, conflicts or unintended consequences of proposed adaptation strategies. 

### 2.6. Step 6: Decision-Making

It is recommended that a final “Climate Change and Health Adaptation Strategies Report” is disseminated to all participants and key decision-makers responsible for the implementation of the potential adaptation strategies. The report should include an overview of the process and a summary of key outcomes including:

- A clear health impact statement including the final risk rankings and vulnerabilities
- Key adaptation actions, especially for priority risks and vulnerable groups

It is recommended that adaptation strategies are provided in a summary table with impacts ordered from the highest to lowest level of risk. An example from the WA study, in which heatwaves were assessed as an extreme risk level, is shown in Table 8 [12]. This table is completed for each of the eight categories of adaptation. The process is repeated for other impacts, with a clear distinction made between different risk levels. 

**Table 8:** Potential Adaptation Strategies and Action Plan for Heatwaves.

Strategies		Actions	Lead Government Agencies	Support Agencies
*Heatwaves: Risk Level = Extreme*				
**1. Legislative or regulatory**				
Heat Event Response Plan	Extend state emergency plan to include heatwaves		Health, Emergency Services	Housing Planning Aged Care
Limit power use in emergency periods	Sectors to discuss feasibility		Energy, Health	Planning Local Government
Regulations for minimum energy efficiencies in homes	Expand energy star codes to existing homes.		Housing, energy	Building industry Businesses
**2. Public education and communication** *(complete for all 8 categories)*				

Table 8 summarises the key information with respect to risk, potential actions and the responsible sectors. Resulting recommendations should include:

- Strategic Direction—incorporation of climate change adaptation strategies into key sustainable development and health plans
- Government Responses—identify lead and support stakeholders particularly with respect to high risk impacts and ensure that high level management are aware of the report and the role of their sector in ensuring appropriate responses to climate change. The health sector should take a lead role by increasing cross-sectoral awareness of connections between health and climate and encouraging appropriate actions to protect the health of the community.
- Community Involvement—education programs regarding the potential health impacts of climate change and the key role of local government or councils with respect to community education.
- Key Activities/Projects—outline the specific projects required. Include cross-cutting measures that have the potential to affect multiple impacts or reduce multiple vulnerabilities. Highlight the potential need for more detailed assessment by other sectors with respect to their role in addressing high priority health risks.

If the project team has access to information that provides an indication of costs associated with recommendations this can also be included in the final report.

## 3. Discussion

The process presented here is intended to assist stakeholders who wish to undertake a HIA of climate change in their region. The use of the established HIA framework complemented with terminology and tools designed specifically for the issue of climate change provides users with a solid foundation to tackle a complex and sometimes overwhelming issue. In particular, the series of working tables incorporate background information on climate and health that provide the Project Team with a significant level of support to direct and plan a HIA of climate change. 

It is clear that the effect of climate change on the health and well-being of people in different locations will be highly variable and to that end, users are encouraged to amend aspects of the process as required. Such adjustments can be considered throughout the process, but should ideally be addressed during the scoping step. The feasibility and effectiveness of adaptation strategies will also be strongly influenced by local circumstances and the delivery of many strategies will sit outside the realm of the health sector. Application of the process to different settings will raise specific questions or suggestions for improvements which are welcomed by the authors.

Use of the framework in several locations has highlighted that limitations in the current state of knowledge can hinder the ability to assess the extent of health impacts related to climate change. This limitation is more a reflection of the complexity of the relationships between climate and health, as well as resource and data limitations, than the framework itself. The identification of gaps in current knowledge is an important outcome of the process that can help to direct future research and data collection. A potential limitation of the process is that workshop participants may not be aware of all relevant information required for a thorough assessment. This limitation is minimised by careful selection and recruitment of the workshop participants. In addition, if participants in the first workshop believe that key stakeholders are absent, further recruitment by the Project Team is possible. Disseminating the interim and final reports to as broad an audience as possible also helps to identify other information that should be considered. 

The requirement for inclusion of stakeholders from multiple disciplines can prove challenging, particularly the need for attendance at up to three workshops. It is envisaged that as the framework is applied to various locations, dissemination of the results provides other Project Teams with the opportunity to review outcomes with respect to their own location and circumstances. This information could potentially reduce the time required for workshops and enable alternative formats such as webinars or virtual workshops. The balance between information needs and available resources and expertise will need to be a key consideration of the Project Team. 

The process is intended to inform decision-making on adaptation planning to address health impacts of climate change. While the final report is critical in terms of communicating the key findings, the interactions between multiple stakeholders that occur throughout the process are paramount. The advances in understanding across multiple sectors of the interactions between people, the environment and climate can result in management improvements across a range of issues. 

## 4. Conclusions

There is widespread agreement that human health and well-being will be influenced by climate change in many ways. It is apparent that an effective response to health impacts of climate change will require the involvement of multiple sectors. Processes that encourage a collaborative approach will contribute to more effective and sustainable management plans that protect the health and well-being of current and future generations. The application of tools tailor-made for climate change, within a HIA framework, supports the need for a collaborative approach. The final recommendations are intended to ensure that decision-makers, who are often required to weigh up multiple factors and interests, integrate evidence regarding health outcomes of climate change into their decision-making process. 

Climate change adaptation will evolve over the coming decades and it is important that as new evidence and experiences emerge, these are incorporated into planning processes. It is envisaged that the application of this framework will assist communities, governments and decision-makers to develop short, medium and long term plans to protect and improve human health and well-being in the face of a changing climate. 
